# Development of Novel Fluticasone/Salmeterol/Tiotropium-Loaded Dry Powder Inhaler and Bioequivalence Assessment to Commercial Products in Rats

**DOI:** 10.3390/pharmaceutics17010103

**Published:** 2025-01-14

**Authors:** Hyukjun Cho, Hyunji Lee, Duhyeong Hwang

**Affiliations:** 1 College of Pharmacy, Keimyung University, Daegu 42601, Republic of Korea; 2 College of Pharmacy, Kyungsung University, Busan 48434, Republic of Korea; hlee@ks.ac.kr

**Keywords:** fluticasone, salmeterol, tiotropium, dry powder inhaler, aerodynamic particle size distribution, dissolution, pharmacokinetics, bioequivalence

## Abstract

**Background**/**Objectives**: Inhaler devices have been developed for the effective delivery of inhaled medications used in the treatment of pulmonary diseases. However, differing operating procedures across the devices can lead to user errors and reduce treatment efficacy, especially when patients use multiple devices simultaneously. To address this, we developed a novel dry powder inhaler (DPI), combining fluticasone propionate (FP), salmeterol xinafoate (SX), and tiotropium bromide (TB) into a single device designed for bioequivalent delivery compared to existing commercial products in an animal model. **Methods**: The micronized FP/SX/TB-loaded capsule was prepared by sieving, blending, and filling capsules. Capsule suitability of the drugs was investigated from the comparison of the stability of drugs within various capsule formulations to that of commercial products. The particle size of the drugs was adjusted using spiral air jet milling, and the ratio of lactose hydrate carriers was optimized by comparing the aerodynamic particle size distribution (APSD) with that of commercial products. To investigate the bioequivalence of micronized FP/SX/TB-loaded DPI to commercial products, the dissolution profile of FP/SX/TB particles and pharmacokinetics in rats were evaluated and compared to commercial products. **Results**: Capsules with hydroxypropyl methylcellulose (HPMC) without a gelling agent showed superior stability of the drugs compared to commercial products. The deposition pattern was influenced by the particle size of the drugs, and fine particle mass exhibited a significant correlation with the amount of fine carrier. Micronized FP/SX/TB-loaded DPI gave a similar APSD and dissolution profile compared to the commercial products and showed dose uniformity by the DPI device. Furthermore, micronized FP/SX/TB-loaded DPI exhibited bioequivalence to commercial products, as evidenced by no significant differences in pharmacokinetic parameters following intratracheal administration in rats. **Conclusions**: A novel triple-combination DPI containing FP/SX/TB was successfully developed, demonstrating comparable pharmacological performance to commercial products. Optimized FP/SX/TB-loaded DPI with HPMC capsule achieved bioequivalence in rat studies, suggesting its potential for improved patient compliance and therapeutic outcomes. This novel single-device DPI offers a promising alternative for triple therapy in pulmonary diseases.

## 1. Introduction

Chronic obstructive pulmonary disease (COPD) is a progressive lung disease with respiratory symptoms, such as shortness of breath, dyspnea, mucinous cough, and exacerbations due to airflow obstruction [1]. The estimated prevalence of COPD in 2019 was approximately 10% among the population aged 30–79, and COPD was ranked as the fourth leading cause of global disability-adjusted life years in the 50–74 age group [2]. According to current guidelines for the pharmacological treatment of patients with COPD, mono/combi-therapy with long-acting β2 agonist (LABA) and long-acting muscarinic antagonist (LAMA) is recommended [3]. For the treatment of patients with a high risk of exacerbation or blood eosinophils, triple inhalation therapy with inhaled corticosteroids (ICS) + LABA + LAMA is recommended [4]. The triple therapy showed notable therapeutic outcomes in reducing exacerbation risk and improving lung function in patients with moderate to very severe COPD [5,6].

Inhalation therapy for pulmonary disease is conducted using devices to deliver accurate doses into airways. Almost all inhaler devices have their own preparation, handling, and operating procedures for optimal use. However, even though the proper use of the device is crucial for the effective treatment of inhalation therapy, handling errors are a problem in patients who are prescribed multi-device [7]. Therefore, the development of a combination inhaler delivered through a single device is necessary to improve patient compliance and therapeutic outcomes. The commercial products containing ICS + LABA + LAMA, such as Trimbow^®^ (beclometasone dipropionate/formoterol fumarate/glycopyrronium bromide), Breztri^®^ aerosphere (budesonide/formoterol fumarate/glycopyrronium bromide), and Trelegy^®^ Ellipta (fluticasone furoate/vilanterol trifenatate/umeclidinium bromide) have been developed [8]. However, the fluticasone propionate (FP)/salmeterol xinafoate (SX)/tiotropium bromide (TB) combination inhaler, which is widely prescribed and offers significant clinical benefits for COPD patients, has not been developed yet [9,10]. This triple combination showed improved pulmonary function compared to mono- and dual-therapy, without an increase in adverse events relative to mono- and dual-therapy [11].

The most widely used inhalers are dry powder inhalers (DPIs) and pressurized metered dose inhalers (pMDIs) [12]. Trimbow^®^ and Breztri^®^ aerospheres are pMDIs, while Trelegy^®^ Ellipta is the only DPI. Due to the use of propellant, proper coordination between inhalation and hand actuation is required when using pMDI. However, this can be difficult for some patients, leading to ineffective medication, increased hospital visits, and poor quality of life [13]. Compared to pMDIs, due to the breath-actuation mechanism, DPIs are easier to use and thus are the most widely prescribed inhalers for the treatment of asthma and COPD [14].

In this study, a novel DPI containing FP (ICS), SX (LABA), and TB (LAMA) has been developed to improve patient compliance and therapeutic outcomes for asthma and COPD patients. The effect of capsule components on stability, particle size of the drugs, and carrier ratio on aerodynamic particle size distribution (APSD) was evaluated. After preparation of micronized FP/SX/TB-loaded DPI, its APSD, dissolution profile of drug particle, and pharmacokinetics of micronized FP/SX/TB-loaded DPI in rats were compared with those of the commercial products.

## 2. Materials and Methods

### 2.1. Materials

FP, SX, and TB were kindly acquired from Hanmi Pharm. Co. (Hwasung, Republic of Korea). Lactose hydrates with various particle sizes (Repitose ML006, Repitose SV003, and Repitose SV010; abbreviated as “ML006,” “SV003,” and “SV010,” respectively) were supplied from DFE Pharma (Goch, Germany), with particle sizes of 17 μm for ML006, 61 μm for SV003, and 109 μm for SV010. Size 3 (15.8 mm × 5.8 mm) hard gelatin capsules containing different ingredients (Embo caps^®^ and Embo caps^®^ PEG) were obtained from Suheung Capsule Co. (Osong, Republic of Korea). Size 3 hard hydroxypropylmethylcellulose (HPMC) capsules containing various ingredients were obtained from various manufacturers: Embo caps^®^ VG (Suheung Capsule Co., Osong, Republic of Korea), Quali-V-I (Qualicaps Co., Nara, Japan), Vcaps^®^ and Vcaps plus^®^ (Capsugel Co., Greenwood, SC, USA). The inhaler device (RS01) is supplied by Plastiape SpA (Osnago, Italy). The commercial products, Seretide Diskus^®^ containing 250 μg of FP and 72.5 μg of SX, and Spiriva Handihaler^®^ containing 21.7 μg of TB, which are reference listed drugs in the Republic of Korea, were purchased from GlaxoSmithKline (Seoul, Republic of Korea) and Boehringer Ingelheim (Seoul, Republic of Korea), respectively. All other chemicals used were reagent grade and applied without further purification.

### 2.2. Preparation of Micronized TB via Air Jet Milling

TB with varying particle sizes was produced through air jet milling using an Alpine 50 AS spiral jet mill (Hosokawa, Augsburg, Germany). The drug was supplied into a jet mill at a rate of 10 g/min with injection air and grinding air pressures set at among 1–4 bars.

The particle size of the micronized TB was measured using high-resolution laser diffraction spectroscopy (HELOS; Sympatec, Clausthal-Zellerfeld, Germany). The micronized TB was analyzed using an R1 lens and dispersed in RODOS/M mode with compressed air at 4.5 bar.

### 2.3. Preparation of Micronized FP/SX/TB-Loaded Capsule

Four micronized FP/SX/TB blend formulations were prepared. Each formulation contained consistent amounts of the drug combination and varying amounts of excipients SV003, SV010, and ML006 (Table 1). FP, SX, TB, SV003, and ML006 powders were blended and passed through a 0.25 mm sieve to break up aggregates during the blending. The resulting blend was then combined with SV010 and sieved again through a 0.25 mm screen. The mixture was blended for 30 min at 49 rpm in a tubular mixer (T2F; Willy A Bachofen AG, Muttenz, Switzerland). The blend of FP/SX/TB equivalent to 250 μg of FP, 72.5 μg of SX, and 21.7 μg of TB was loaded into size 3 hard capsules as listed in Table 2.

### 2.4. Effect of Capsule Components on Drug Stability

The effect of capsule components on the stability of FP, SX, or TB was investigated. The main materials and gelling agent of the capsule are described in Table 2. Each capsule was packed in an aluminum–aluminum blister and stored at 60 °C for 2 weeks [15,16]. For comparison, commercial products are stored under the same conditions. Degraded compounds of FP, SX, or TB were analyzed as described in Section 2.5.

### 2.5. Quantification of Degradation Compounds of Drugs

Degradation compounds of FP, SX, or TB were quantified using a high-performance liquid chromatography (HPLC) system (Hitachi, Tokyo, Japan) comprising a 5110 pump, 5430 detectors, a 5310 column oven, and 5210 autosamplers. While FP and SX were analyzed together, TB was assessed using a separate set of analytical conditions. Inertsil^®^ ODS-2 column (4.6 mm × 250 mm, 5 μm) was used to analyze the degradation compounds of FP and SX. The powder, equivalent to 2.5 mg of FP and 0.725 mg of SX, was introduced into 5 mL of a mixture consisting of phosphoric acid, methanol, and distilled water in a ratio of 0.05:89.95:10. The mixture solution was vortexed for 30 min and passed through a 0.45 μm membrane filter. Fifty microliter aliquots of the filtered solution were injected and detected at 228 nm. The ratio of mobile phases A (0.05 M ammonium phosphate monobasic, pH 2.9) and B (acetonitrile) was adjusted in gradient mode, transitioning from 7:3 at 0 min to 2:8 at 60 min. The flow rate of the mobile phase was 1.0 mL/min, and the column temperature was set to 35 °C. The standard curve showed excellent linearity with R^2^ more than 0.999 across the concentration range of 0.25–3 μg/mL for FP and 0.07–8.7 μg/mL for SX, respectively. The Zorbax^®^ SB-3 column (3.0 mm × 150 mm, 3.5 μm) was selected to analyze impurities of TB. Mobile phase C was a pH 3.0 buffer containing sodium methanesulfonate (1 g/L), and mobile phase D was a mixture consisting of methanol, acetonitrile, and mobile phase C in a ratio of 10:40:50. The ratio of mobile phases C and D was adjusted in gradient mode, starting at 90:10 and changing to 25:75 by 30 min. The powder, equivalent to 0.5 mg of TB, was dissolved into 1 mL of mobile phase D and vortexed for 30 min. Fifteen microliter aliquots of the sample solution were injected into the column at 50 °C and monitored at 240 with a flow rate of 1.2 mL/min. The standard curve showed excellent linearity with R^2^ exceeding 0.999 across the concentration range of 0.05–6 μg/mL.

### 2.6. Aerodynamic Particle Size Distribution of Micronized FP/SX/TB-Loaded DPI

APSD of micronized FP/SX/TB-loaded capsules was evaluated using the Anderson Cascade Impactor with pre-separator (ACI; Copley Scientific, Nottingham, UK) and an inhaler device (RS01; Plastiape SpA, Osnago, Italy). To ensure efficient particle capture and prevent re-entrainment, 10 mL of distilled water was placed in the pre-separator, and the collection plates were coated with a mixture consisting of glycerol, polyoxyethylene lauryl ether, and ethanol in a weight ratio of 100:3:20. The size 3 hard capsule was inserted and punctured in an inhalation device. The drug blend in the capsule was dispersed by drawing 4.0 L of air, with flow rates adjusted to 60 L/min, and 10 actuations were performed for size distribution measurement. Deposited drug blends on each collection plate were individually rinsed and collected using a mixture composed of 0.01 M HCl, edetate disodium dihydrate, and methanol in a weight ratio of 300:0.075:700. The solution was filtered through a 0.45 μm PVDC membrane filter, and its concentration was analyzed using an HPLC system. FP and SX were analyzed simultaneously, while TB was analyzed using different analytical conditions, as follows.

As the mobile phase for FP and SX, a mixture of methanol, acetonitrile, and distilled water (50:16:34, volume ratio) with 0.6% ammonium acetate was used. Inertsil^®^ ODS-3 column (4.6 mm × 150 mm, 5 μm) was placed in a column oven, which was maintained at a temperature of 40 °C. The flow rate of the mobile phase was 1.7 mL/min, and the sample solution (100 μL) was monitored at 228 nm. The linearity of standard curves was confirmed, showing R^2^ more than 0.999 over the range of 0.05–30 μg/mL for FP and 0.05–7 μg/mL for SX, respectively.

As the mobile phase for TB, 1.25 g of sodium 1-heptansulfonate was dissolved in distilled water, and the pH was adjusted to 3.2 with phosphoric acid. The solution was mixed with acetonitrile in a volume ratio of 700:330 and used as a mobile phase, with the flow rate set at 1.7 mL/min. The Spherisorb^®^ ODS2 column (4.0 mm × 125 mm, 5 μm) was installed in a column oven, with the temperature set to 40 °C. The injection volume of the sample solution was 100 μL and monitored at 240 nm. The linearity of standard curves was confirmed, showing R^2^ more than 0.999 across the concentration range of 0.03–3 μg/mL. The fine particle mass (FPM) was calculated as the sum of the drugs on stages 2–6 representing fine particles less than 5 μm.

### 2.7. Delivered Dose Uniformity of Micronized FP/SX/TB-Loaded DPI

The delivered dose uniformity (DDU) of the micronized FP/SX/TB-loaded DPI was determined using a DDU sampling apparatus (Copley Scientific, Nottingham, UK). The airflow rate and duration time of each actuation were adjusted to induce a 4.0 kPa pressure drop and 2.0 L of air. The amount of FP, SX, and TB from each of the 10 separate capsules was individually determined by HPLC, described in Section 2.5.

### 2.8. Dissolution Profile of Micronized FP/SX/TB-Loaded DPI

To collect the fine particles less than 5 μm on a glass fiber filter, abbreviated ACI was used [17,18]. The micronized FP/SX/TB blend was dispersed at a flow rate of 60 L/min, and 4.0 L of air was withdrawn. The glass fiber filter with collected fine particles was assembled into a filter holder (Copley Scientific, Nottingham, UK). The dissolution profiles of micronized FP/SX/TB-loaded DPI were assessed using a USP dissolution tester equipped with a paddle agitator (PTWS-1220; Pharma test apparatus AG, Darmstadt, Germany). The filter holder was placed in a dissolution vessel, and 500 mL of simulated lung fluid was used as dissolution media [19]. The dissolution media was maintained at a temperature of 37 ± 0.5 °C with an agitation speed of 75 rpm. At a predetermined time, 2 mL of the media was collected and diluted with 0.1 M HCl, edetate disodium dihydrate, and methanol to achieve a ratio of 900:50:0.075:50. The diluted solution was filtered through a 0.45 μm PVDC membrane filter and analyzed by HPLC as described in Section 2.5.

### 2.9. Pharmacokinetic Evaluation

#### 2.9.1. Administration to Rats

The pharmacokinetic studies were performed with the approval of the Institutional Animal Care and Use Committee (IACUC) at the Hanmi Research Center. Twelve male Sprague-Dawley rats (270–330 g) were randomly divided into two groups and fasted overnight. In each group, the rats were administered powder via the pulmonary route at a dose of 250 μg/kg FP, 72.5 μg/kg SX, and 21.7 μg/kg TB. An insufflator device (DP-4; Penn-Century Inc., Philadelphia, PA, USA) was used for intratracheal administration, delivering a micronized FP/SX/TB blend by discharging 4 mL of air using DPI. The blood samples (approximately 0.5 mL) were drawn from the jugular vein and collected into microtubes containing heparin (1000 IU/mL, 3 μL). The blood samples were promptly centrifuged at 13,000× *g* for 3 min at 4 °C using a centrifuge (5415C; Eppendorf, Hauppauge, NY, USA). The plasma was separated and kept at −80 °C until further analysis.

#### 2.9.2. Plasma Sample Preparation and Analysis

To determine plasma concentrations of FP and SX, the internal standard solution containing fluticasone propionate-d3 and salmeterol-d3 was prepared at concentrations of 3000 ng/mL and 600 ng/mL, respectively. The plasma samples (50 μL), internal standard (50 μL), and ethyl ether (900 μL) were placed in a microtube and mixed for 1 min using a vortex mixer. The mixture was centrifuged at 13,000× *g* for 3 min. The supernatant (100 μL) was mixed with acetonitrile (100 μL) for 1 min and centrifuged at 13,000× *g* for 3 min. The supernatant (20 μL) was analyzed by mass spectrometry (LC–MS/MS, API 5000, AB/MDS SCIEX, Concord, ON, Canada) coupled with an HPLC system (1200 series, Agilent Technologies, Inc., Palo Alto, CA, USA). The column was a C18 column (YMC-Pack Pro, 150 mm × 2.0 mm, 5 μm). The mobile phase consisted of a mixture of acetonitrile and 5 mM ammonium acetate (90:10) with a flow rate of 0.2 mL/min. The turbo ion spray ionization positive mode and multi-reaction monitoring (MRM) detection were used. Ion spray voltage was set to 5.5 kV. The collision energy and collision cell exit potential energy were 21 V and 22 V for FP and 33 V and 32 V for SX, respectively.

To quantify the plasma concentration of TB, the plasma samples (50 μL) were mixed with tiotropium-d3 solution (500 ng/mL, 30 μL) as an internal standard. Methanol (400 μL) was added and mixed for 1 min. To separate the supernatant, the mixture was centrifuged at 13,000× *g* for 3 min. The supernatant (100 μL) and distilled water containing 0.1% formic acid (100 μL) were mixed for 1 min and centrifuged at 13,000× *g* for 3 min. The supernatant (5 μL) was analyzed by LC–MS/MS (TQ-S, Waters, Massachusetts, MA, USA). The column oven temperature was maintained at 25 °C, and the phenyl column (Zorbax SB-phenyl, 100 mm × 2.1 mm, 1.7 μm) was assembled. The flow rate of the mobile phase, consisting of a mixture of methanol and 5 mM ammonium acetate with 0.1% formic acid in a ratio of 50:50, was 0.25 mL/min. As a detector mode, electrospray ionization (positive ion mode) and MRM mode were set. The cone voltages, capillary voltages, and collision energy were 45 V, 3.0 kV, and 23 V, respectively.

## 3. Results and Discussion

### 3.1. Effect of Capsule Components on Drug Stability

**Figure 1:**
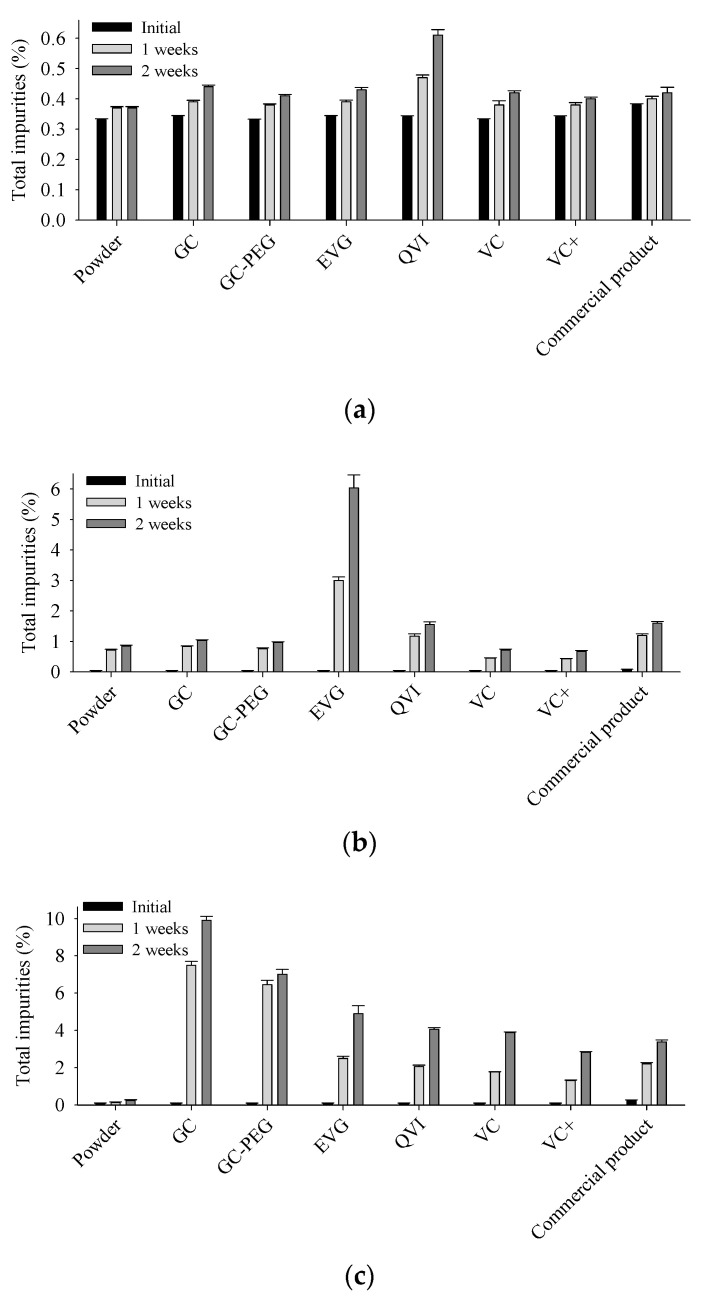
Total percentage of degraded compounds from (**a**) FP, (**b**) SX, or (**c**) TB in capsules with various components at 60 °C for 2 weeks. Each value represents the mean ± S.D. (*n* = 3).

In this study, a novel triple combination DPI containing FP, SX, and TB was prepared as a dry powder in capsule form. In capsule-based DPIs, the chemical components of the capsules significantly influence the aerodynamic performance and stability of drugs within the capsule [20]. Thus, the degraded compounds from FP, SX, or TB in various capsules (Table 1) at 60 °C for 2 weeks (accelerated condition) were analyzed (Figure 1). The total degraded compound of FP was less than 0.7% of the initial amount, indicating better stability compared to SX and TB. Total degraded compounds of SX and TB increased notably in EVG and GC capsules, respectively, in accelerated conditions. For SX, the capsule showing both the highest and the lowest increase in the degraded compound was the HPMC capsule. In contrast, for TB, higher degraded compounds were observed in gelatin-based capsules (GC and GC-PEG) compared to HPMC capsules. Gelatin and HPMC capsules differ significantly in water content (13–15% vs. 4–6%) and the presence of a gelling agent in HPMC capsules, which varies by manufacturer. These differences substantially affect the increase in degraded compounds [21]. Excess water content promotes drug degradation, particularly hydrolysis in the case of TB; therefore, choosing a capsule with low water content was essential [22,23]. SX mainly degrades in acidic conditions, and HPMC capsules containing a gelling agent create a weak, acidic environment [24,25]. Therefore, VC+, which contains no gelling agent or other ingredients, may exhibit better stability than other HPMC capsules and commercial products. Based on the overall results, VC+, which exhibited the lowest increase in impurities for FP, SX, and TB, was selected as the capsule shell for further evaluation.

### 3.2. Micronized TB via Air Jet Milling

The particles with an aerodynamic size of less than 5 μm are considered efficiently inhalable into the deep lung [26]. In this study, the particle sizes of FP and SX powders were less than 5 μm without the air jet milling process. However, the particle size of TB from the manufacturer was 6.1 ± 0.8 μm. To reduce the size of TB to less than 5 μm, a spiral jet mill process was applied (Table 3). In the spiral jet mill, TB yield decreased when the injection air pressure and grinding air pressure were similar [27]. To achieve a TB yield of more than 85%, the injection air pressure was set at least 1 bar higher than the grinding air pressure. As a result, the particle size of TB was decreased as the grinding air pressure increased (condition I, 3.1 ± 0.3 μm; condition II, 2.5 ± 0.1 μm; condition III, 1.4 ± 0.3 μm).

**Figure 2:**
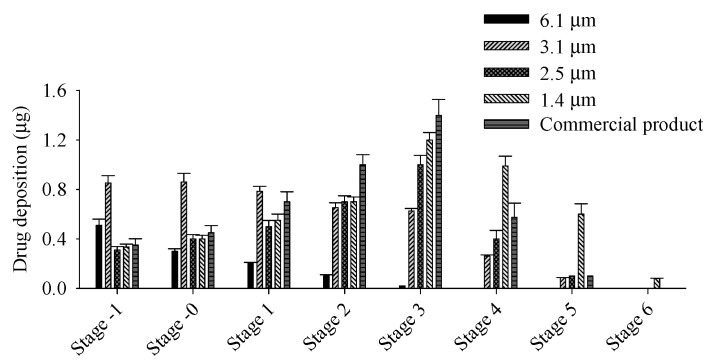
Effect of particle size of TB on the APSD. Each value represents the mean ± S.D. (*n* = 3).

The aerodynamic size of particles is crucial and significantly impacts pulmonary delivery [28,29]. Therefore, the effect of particle size of TB on the APSD was investigated using ACI, as described in Section 2.6. Micronized FP/SX/TB blends with different particle sizes of TB were prepared according to composition I (Table 1), and their APSD was compared with those of commercial products (Figure 2). The drug deposition at each stage was significantly affected by the particle size of TB. At a flow rate of 60 L/min, the cut-off diameters for each stage are as follows: 8.6 μm for stage −1, 6.5 μm for stage 0, 4.4 μm for stage 1, 3.2 μm for stage 2, 1.9 μm for stage 3, 1.2 μm for stage 4, 0.55 μm for stage 5, and 0.26 μm for stage 6 [29]. For micronized FP/SX/TB-loaded DPI with larger TB particles, deposition increased in the upper stages of the ACI, whereas for DPIs with smaller TB particles, deposition increased in the lower stages, indicating improved deposition in the deep lung [30,31]. Regarding the TB deposition pattern, micronized FP/SX/TB-loaded DPI with TB particle sizes of 6.1 and 3.1 μm were primarily deposited on stages −1 and 0, which have larger cut-off diameters. In contrast, TB with a particle size of 1.4 μm showed the highest deposition in stages 3 and 4, indicating efficient lung delivery. For the commercial product, Spiriva^®^, maximum deposition was obtained on stage 3, with significantly higher amounts compared to stages 2 and 4 (*p* < 0.05). The deposition site of particles is determined by their aerodynamic particle size, which in turn affects pulmonary absorption and drug efficacy [32]. Therefore, to achieve bioequivalence with commercial products, the deposition pattern of micronized FP/SX/TB-loaded DPI must be carefully considered [33]. Based on the result that micronized FP/SX/TB-loaded DPI with a TB particle size of 2.5 μm showed maximum deposition in stage 3 and significantly higher amounts compared to stages 2 and 4, the TB particle size was set to 2.5 μm to achieve a similar deposition pattern to the commercial product. Consequently, the process parameters of the spiral jet mill were optimized to an injection air pressure of 3 bar and a grinding air pressure of 2 bar, referred to as condition II (Table 3).

### 3.3. Aerodynamic Performance of Micronized FP/SX/TB-Loaded DPI

**Figure 3:**
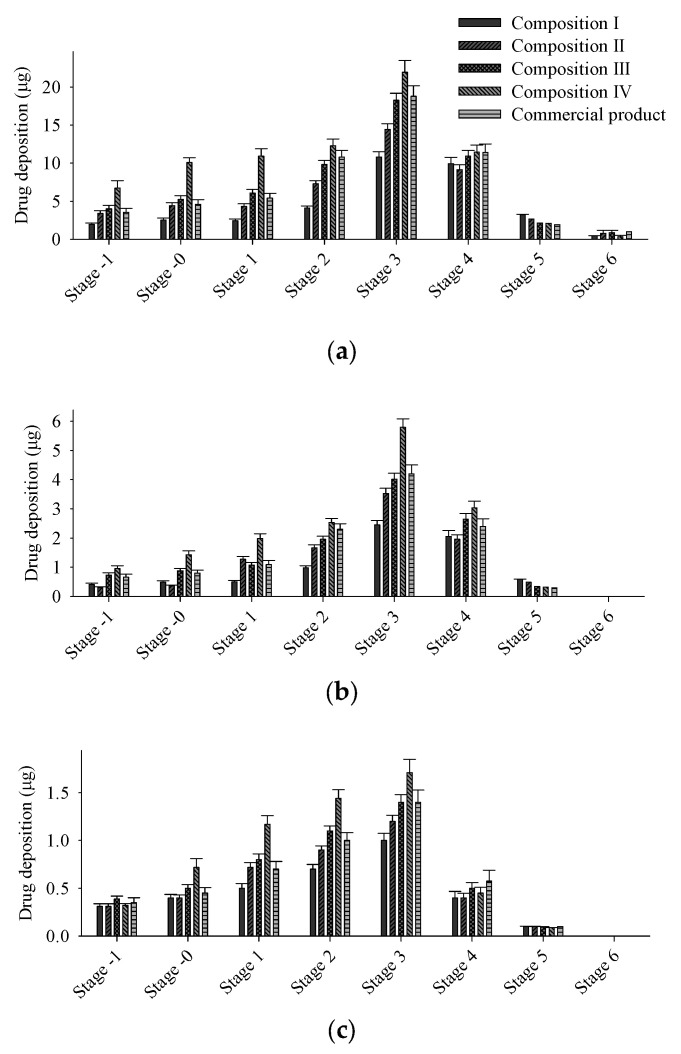
Effect of excipients on the APSD of micronized FP/SX/TB-loaded DPI: (**a**) FP; (**b**) SX; (**c**) TB. Each value represents the mean ± S.D. (*n* = 3).

To assess the impact of lactose hydrate carrier ratios on FPM, various micronized FP/SX/TB blends were prepared with differing lactose hydrate ratios. Then, particle size and aerodynamic performance were compared across formulations (Table 4). The total amount of micronized FP/SX/TB blend in each capsule was approximately 12.5 mg, comparable to a commercial product, Seretide Diskus^®^ [34]. In this study, three grades of lactose hydrate were used with particle sizes of 109 μm for SV010, 61 μm for SV003, and 17 μm for ML006 [35]. Blends containing more than 3.6 mg of ML006 exhibited poor flowability and were excluded from the evaluation. The overall deposited amount of the drugs in stages −1 through 6 increased with a higher amount of ML006 (Figure 3). Furthermore, the FPM was significantly influenced by the amount of ML006 (Table 3). The large carriers, such as SV010 and SV003, were used in a ratio of 1:1, while the amount of ML006 was adjusted to control FPM [36]. The micronized carrier enhances efficient lung delivery, while a coarse carrier is also required to improve flowability for automated capsule filling [37,38]. The fine carrier (ML006) with high surface energy forms strong adhesive interactions with the drug during blending, resulting in agglomerates [39,40]. These agglomerates adhere to the surfaces of the large carriers (SV003 and SV010). During inhalation, the agglomerates detach more easily from the large carriers, enhancing lung delivery efficiency [40]. Moreover, the fine carrier causes drugs to compete for high-energy binding sites on a large carrier surface, forcing the drugs to bind to low-energy binding sites [41]. This competitive binding results in improved detachment from the large carrier, enhancing dispersion efficiency [42].

**Figure 4:**
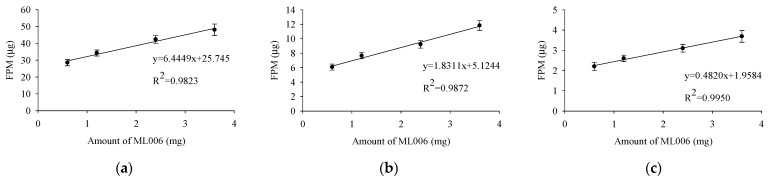
Correlation between the amount of ML006 and the FPM of micronized FP/SX/TB-loaded DPI (**a**) FP; (**b**) SX; (**c**) TB. Each value represents the mean ± S.D. (*n* = 3).

The correlation between the amount of ML006 and FPM was also investigated (Figure 4). The FPM showed a linear correlation (R^2^ > 0.98) with the ML006 content, indicating that the FPM of micronized FP/SX/TB-loaded DPI can be precisely controlled by adjusting the amount of ML006. The fine carrier that demonstrates a linear relationship with FPM is the cumulative fraction with particle sizes below 30 μm [43]. Since the particle size of ML006 is 17 μm, and SV003 and SV010 are significantly larger than 30 μm, the amount of ML006 showed a strong correlation with FPM. According to guidance for bioequivalence approaches of orally inhaled drug products, the equivalence criteria of APDS is ± 15% [44,45]. Based on the APSD results, the composition III demonstrated FPM and aerodynamic particle deposition patterns equivalent to commercial products, with FPM values of 42.4 ± 2.3 vs. 43.9 ± 3.4 μg for FP, 9.2 ± 0.5 vs. 9.2 ± 0.8 μg for SX, 3.1 ± 0.2 vs. 3.1 ± 0.3 μg for TB, respectively. Therefore, composition III was determined to be the optimum formulation for micronized FP/SX/TB-loaded DPI.

Furthermore, the DDU of micronized FP/SX/TB-loaded DPI was also evaluated using the DDU sampling apparatus. DDU is a critical quality attribute of inhalers, ensuring consistent administration of drugs through the inhaler [46]. According to USP, the acceptance criteria of DDU require that the delivered dose from each capsule be within 75–125% of the target delivered dose [47]. The DDU of micronized FP/SX/TB-loaded DPI demonstrated excellent dose uniformity and met the USP criteria, with results of 157.4 ± 3.9 μg (94.8~103.4%) for FP, 33.8 ± 1.7 μg (92.2~108.8%) for SX, and 11.1 ± 0.9 μg (87.5~108.3%) for TB, respectively.

### 3.4. Dissolution of Micronized FP/SX/TB-Loaded DPI

**Figure 5:**
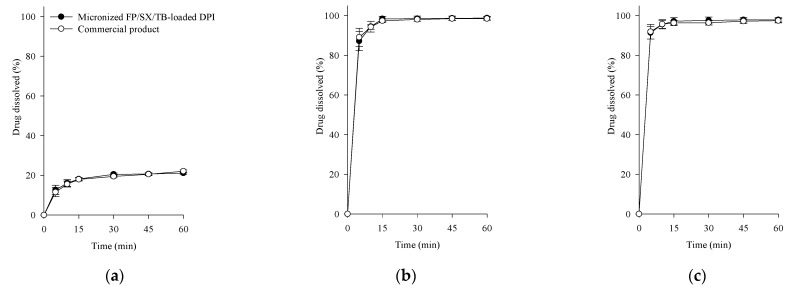
Comparative dissolution profiles of FPM from micronized FP/SX/TB-loaded DPI (Composition III) and commercial products: (**a**) FP; (**b**) SX; (**c**) TB. Each value represents the mean ± S.D. (*n* = 6).

Pulmonary absorption and drug performance are highly influenced by solubility and dissolution [48,49]. To assess bioequivalence, the fine particles less than 5 μm were collected using an abbreviated ACI, and their dissolution profiles were compared (Figure 5). FP exhibited low dissolution, whereas SX and TB showed more than 85% dissolved within 15 min. FP is the biopharmaceutical classification system class II drug with limited solubility, showing a maximum dissolution of approximately 20% [50]. The micronized FP/SX/TB-loaded DPI and commercial products showed no significant difference in their dissolution profiles. Furthermore, the difference factor (*f*_1_) and similarity factor (*f*_2_) were calculated:

(1) $$f_{1}=\left[\frac{\sum |R_{t}-T_{t}|}{\sum R_{t}}\right]\times 100$$

(2) $$f_{2}=50\times log\left[\frac{100}{\sqrt{1+\frac{\sum {\left(R_{t}-T_{t}\right)}^{2}}{n}}}\right]$$

where *n* is the number of time points, and R_t_ and T_t_ are the mean percent of drug dissolved at time t for the commercial products and micronized FP/SX/TB-loaded DPI, respectively [51,52]. The values of 0 < *f*_1_ < 15 and 50 < *f*_2_ < 100 are considered indicative of the similarity between the two dissolution profiles [53]. The calculated f_1_ values were 4.1 for FP, 1.1 for SX, and 0.5 for TB, while the corresponding *f*_2_ values were 95.5, 89.8, and 96.7, respectively. These results indicate that the dissolution profiles of the fine particles from micronized FP/SX/TB-loaded DPI and commercial products were equivalent.

### 3.5. Pharmacokinetics

**Figure 6:**
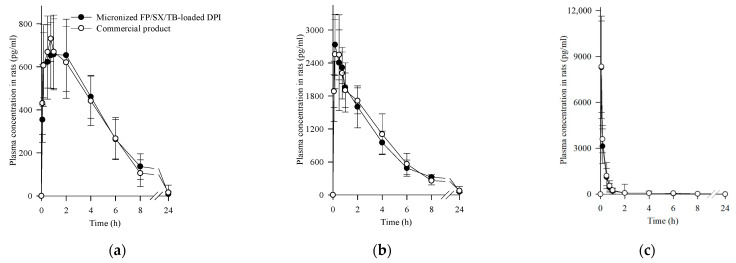
Plasma concentration-time profiles of FP, SX, and TB after the intratracheal administration of micronized FP/SX/TB-loaded DPI (Composition III) and commercial products to rats: (**a**) FP; (**b**) SX; (**c**) TB. Each value represents the mean ± S.D. (*n* = 6).

Similar to oral dosage forms, pharmacokinetic comparisons are necessary to demonstrate bioequivalence between inhaled drug products [54,55]. Following intratracheal administration of micronized FP/SX/TB-loaded DPI and commercial products, plasma concentrations of drugs and the commercial products were analyzed and are presented in Figure 6. Pharmacokinetic parameters are summarized in Table 5.

The plasma concentrations of the drugs from micronized FP/SX/TB-loaded DPI and the commercial products showed no significant difference at each time point (*p* > 0.05) [56]. As both are DPIs, the maximum plasma concentration was reached in a short time, indicating rapid absorption, with the T_max_ of each drug following the order TB < SX < FP [57]. Specifically, TB showed a particularly high C_max_ and rapid elimination. FP exhibited a relatively delayed and variable T_max_ compared to TB and SX, which is likely attributable to the limited solubility of FP [58,59]. Furthermore, there were no significant differences in pharmacokinetic parameters, such as AUC, C_max_, and T_max_. Therefore, micronized FP/SX/TB-loaded DPI showed bioequivalence to the commercial products in rats.

## 4. Conclusions

In summary, this study successfully developed a stable and bioequivalent triple-combination DPI for pulmonary drug delivery. The optimized formulation, utilizing HPMC capsules without gelling agent and a defined ratio of excipients (SV006:SV010:ML006 at 4.9:4.9:2.4), achieved comparable aerodynamic particle size distribution, fine particle dissolution, and delivered dose uniformity to existing commercial products. Critically, in vivo pharmacokinetic assessments in rats confirmed bioequivalence, supporting the potential of this single-device, triple-drug DPI to improve patient compliance and therapeutic outcomes compared to current treatment options.

## Figures and Tables

**Table 1:** Composition of micronized FP/SX/TB blend. Each value represents the mean ± S.D. (*n* = 3).

Ingredients	Composition I	Composition II	Composition III	Composition IV
FP	0.25	0.25	0.25	0.25
SX	0.0725	0.0725	0.0725	0.0725
TB	0.0217	0.0217	0.0217	0.0217
SV003	5.8	5.5	4.9	4.3
SV010	5.8	5.5	4.9	4.3
ML006	0.6	1.2	2.4	3.6
Total weight (mg)	12.5442	12.5442	12.5442	12.5442

**Table 2:** Components and gelling agent of capsules.

Capsules	Brand Name	Main Materials	Gelling Agent	Manufacturer
GC	Embo caps^®^	Gelatin	None	Suheung
GC-PEG	Embo caps^®^ PEG	Gelatin	None	Suheung
EVG	Embo caps^®^ VG	HPMC	Amidated pectin	Suheung
QVI	Quali-V-I^®^	HPMC	Carrageenan	Qualicaps
VC	Vcaps^®^	HPMC	Gellan gum	Capsugel
VC+	Vcaps plus^®^	HPMC	None	Capsugel

**Table 3:** Air jet mill conditions and the resulting particle size of TB. Each value represents the mean ± S.D. (*n* = 3).

Parameter	I	II	III
Injection air pressure (bar)	2	3	4
Grinding air pressure (bar)	1	2	3
Particle size (μm)	3.1 ± 0.3	2.5 ± 0.1	1.4 ± 0.3

**Table 4:** FPM of micronized FP/SX/TB blend in VC+ capsule. Each value represents the mean ± S.D. (*n* = 3).

		Composition I	Composition II	Composition III	Composition IV
FPM (μg)	FP	28.5 ± 1.8	34.3 ± 1.8	42.4 ± 2.3	48.1 ± 3.4
	SX	6.1 ± 0.4	7.7 ± 0.4	9.2 ± 0.5	11.8 ± 0.7
	TB	2.2 ± 0.2	2.6 ± 0.2	3.1 ± 0.2	3.7 ± 0.3

**Table 5:** Pharmacokinetic parameters after the intratracheal administration of micronized FP/SX/TB-loaded DPI (Composition III) and commercial products to rats. Each value represents the mean ± S.D. (*n* = 6).

Drugs		AUC (h·pg/mL)	C_max_ (pg/mL)	T_max_ (h)	t_1/2_ (h)	K_el_ (h^−1^)
FP	Micronized FP/SX/TB-loaded DPI	3857.45 ± 1432.89	657.58 ± 227.16	0.71 ± 0.73	3.58 ± 0.66	0.19 ±0.06
	Commercial product	3837.99 ± 1880.79	730.73 ± 141.02	1.40 ± 1.42	4.15 ± 1.06	0.17 ± 0.06
SX	Micronized FP/SX/TB-loaded DPI	12,608.4 ± 2487.89	2796.53 ± 239.54	0.50 ± 0.00	4.89 ± 1.26	0.14 ± 0.06
	Commercial product	11,691.37 ± 3272.39	2557.60 ± 352.26	0.33 ± 0.18	4.41 ± 1.01	0.16 ± 0.05
TB	Micronized FP/SX/TB-loaded DPI	2753.00 ± 1911.75	8288.30 ± 351.72	0.08 ± 0.00	0.87 ± 0.11	0.81 ± 010
	Commercial products	2590.98 ± 628.43	8343.30 ± 2987.22	0.08 ± 0.00	1.04 ± 0.19	0.75 ± 0.14

## Data Availability

The data presented in this article are contained in the manuscript.
